# Research progress on cellular behavior of CaSR in cardiovascular diseases

**DOI:** 10.3389/fimmu.2025.1672536

**Published:** 2025-10-23

**Authors:** Xinyu Liu, Tao Xie, Javeria Qadir, Shuang Li, Tianshuang Xu, Yang Lu, Xiaotian Ge, Hui Yuan

**Affiliations:** ^1^ School of Basic Medical Sciences, Mudanjiang Medical University, Mudanjiang, China; ^2^ School of Clinical Medicine, Jiamusi University, Jiamusi, China; ^3^ Department of Biosciences, COMSATS University Islamabad, Islamabad, Pakistan; ^4^ Department of Immunology, Mudanjiang Medical University, Mudanjiang, China; ^5^ Department of Gastroenterology, Mudanjiang Traditional Chinese Medicine Hospital, Mudanjiang, China; ^6^ School of Stomatology, Mudanjiang Medical University, Mudanjiang, China

**Keywords:** CaSR, calcium homeostasis, cardiovascular diseases, apoptosis, autophagy, pyroptosis

## 1 Introduction

Cardiovascular diseases (CVDs) remain the leading cause of morbidity and mortality worldwide, posing a major burden on global health system. Despite advancements in diagnosis and therapy, the underlying cellular and molecular mechanisms contributing to the development and progression of CVDs are not fully understood (1). Among them, the dysregulation of calcium signal transduction, as a crucial pathological physiological process, is receiving increasing attention, particularly in terms of calcium signal imbalance and its impact on the survival and function of cardiac cells. Therefore, Calcium-sensing receptor (CaSR) can serve as an important target for the treatment of cardiovascular diseases.

CaSR is a cell surface receptor that functions by sensing the concentration of Ca^2+^ in the extracellular matrix (ECM), thus, serving as a primary regulator of calcium homeostasis. Calcium is an important ion in the cells, highly ubiquitous and an integral intracellular messenger that participates in various biological processes (2–4). The alterations in extracellular Ca^2+^ concentration can affect normal cellular activities and physiological functions of these systems.

Several cardiovascular conditions, including hypertension, atherosclerosis, cardiac hypertrophy, myocardial infarction (MI), and vascular calcification, have been associated with altered CaSR expression or signaling. These associations have spurred growing interest in the therapeutic modulation of CaSR using pharmacological agents such as calcimimetics and calcilytics. While these agents have shown potential in preclinical models, their use in cardiovascular therapy remains limited due to concerns regarding systemic side effects and inconsistent outcomes (5, 6).

Given the expanding body of literature on CaSR’s involvement in cardiovascular health and disease, a comprehensive review of its cellular biological behaviors is timely and necessary. This article aims to summarize current knowledge on CaSR expression and function in cardiovascular cell types, discuss its role in various cardiovascular pathologies, highlight contradictory findings and research gaps, and explore the therapeutic potential of CaSR-targeted interventions. Such insights may help pave the way toward more targeted and effective strategies for managing CVDs.

## 2 CASR structure and its functional significance

According to research (7), the CaSR was initially cloned from bovine parathyroid glands, where investigators identified a high degree of similarity to glutamate receptors.

CaSR is a member of the G protein-coupled receptor (GPCR) family. The receptor possesses a substantial extracellular domain, which encompasses clusters of acidic amino acid residues potentially involved in calcium binding. Structurally, it consists of four parts: (i) a large N-terminal extracellular domain (ECD), (ii) a cysteine-rich domain, (iii) a seven-transmembrane helical domain (TMD), and (iv) a C-terminal tail (8–10). Adjacent to the C-terminus of the signal peptide at the N-terminal extremity of the protein lies a large bilobed structure known as the “Venus Flytrap domain” (VFT domain) (7). The ECD of CaSR is composed of two dimerized lobes, LB1 and LB2, which are separated by a central cavity. Calcium binds within the cleft between the two lobes of each VFT unit, leading to receptor activation. Receptor activation stimulates the intrinsic seven-transmembrane helical domain (7TM) in G protein-coupled homodimers (8, 11).

**Figure 1:**
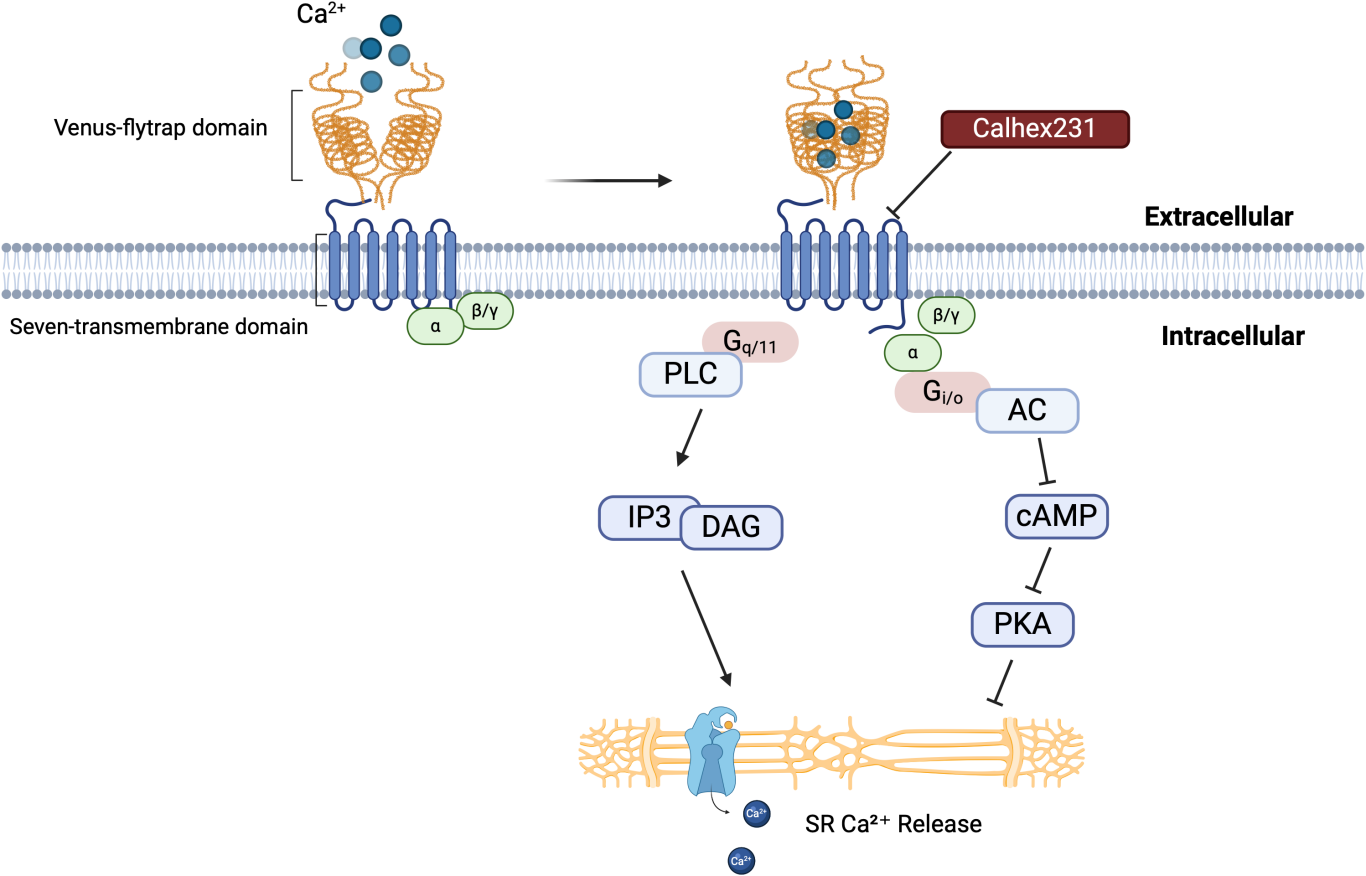
CaSR structure and activation mechanism. The interaction between the extracellular domain of the CaSR, known as the “Venus flytrap,” and calcium ions initiates receptor activation. This activation leads to conformational changes that trigger downstream signaling pathways. Calhex231, a well-characterized inhibitor of CaSR, can prevent this activation process. This figure provides a comprehensive overview of the CaSR activation mechanism and its role in regulating intracellular calcium levels through multiple signaling pathways. A thorough understanding of these mechanisms is essential for clarifying the physiological and pathophysiological roles of CaSR in various cellular processes.

When CaSR is activated, its homodimer binds to various ligands and heterotrimeric modulators (12–15), mediating downstream signaling through G protein to generate biological effects such as G_i/o_ and G_q/11_ (16–19). After binding to G_i/o_ protein, CaSR inhibits the production of cyclic adenosine monophosphate (cAMP) through adenylate cyclase, thereby, suppressing the activation of protein kinase A (PKA), which results in a reduction of Ca^2+^ release from the sarcoplasmic reticulum (20). G_q/11_ activates inositol 1,4,5-trisphosphate (IP3) and diacylglycerol (DAG) pathways together with phospholipase (21, 22), as illustrated in the **Figure 1** below. IP3 regulates the intracellular influx and efflux of Ca^2+^ by controlling calcium channels on the cell surface. Abnormal activation or inhibition of the cAMP signaling pathway plays an important role in the pathogenesis of CVDs. Although the published studies on CaSR have provided potential therapeutic strategies and directions for understanding CaSR signaling mechanisms, nonetheless, they still have limitations. At present, the downstream signaling pathways of CaSR have not been fully understood, and the specific mechanisms of action in different tissues and various pathological conditions have not been fully investigated either. These limitations indicate that in future research, a more in-depth clarification of the pathway mechanisms is still needed.

CaSR plays a pivotal role in regulating extracellular Ca^2+^ homeostasis, influencing numerous physiological and pathophysiological processes across multiple organs, including the heart (23, 24), parathyroid glands (25, 26), kidneys (27–29), bones (30, 31), brain (32), and skin (33, 34). Decreased intracellular Ca^2+^ concentration eventually leads to reduced myocardial contractility and contraction rate. Ca^2+^ signals can potentially modulate cellular processes such as apoptosis, autophagy and pyroptosis, which are closely associated with cardiac diseases, including myocardial ischemia-reperfusion (I/R) injury, heart failure, myocardial fibrosis, and diabetic cardiomyopathy (35–37). The disruption of Ca^2+^ homeostasis can lead to various types of cell death, whereby, CaSR regulates processes such as apoptosis, autophagy, and pyroptosis by sensing changes in Ca^2+^ concentration and the pH. Additionally, it plays an integral role in various cellular aspects such as cell structure, protease activity, and ROS production, thus, influencing tissue remodeling, repair, and disease progression (4, 38–40). Therefore, CaSR emerges as a promising therapeutic target with substantial biological and clinical relevance for disease intervention. The current review aims to explore the emerging roles of CaSR in various cellular processes implicated in CVDs.

## 3 Role of CaSR in various cellular processes

CaSR regulates diverse cellular processes including proliferation, apoptosis, and inflammation. It modulates intracellular calcium signaling, thus, impacting cell differentiation, migration, and oxidative stress responses. Through these pathways, CaSR plays a crucial role in maintaining tissue homeostasis and contributing to disease pathogenesis including CVDs.

### 3.1 Apoptosis

**Figure 2:**
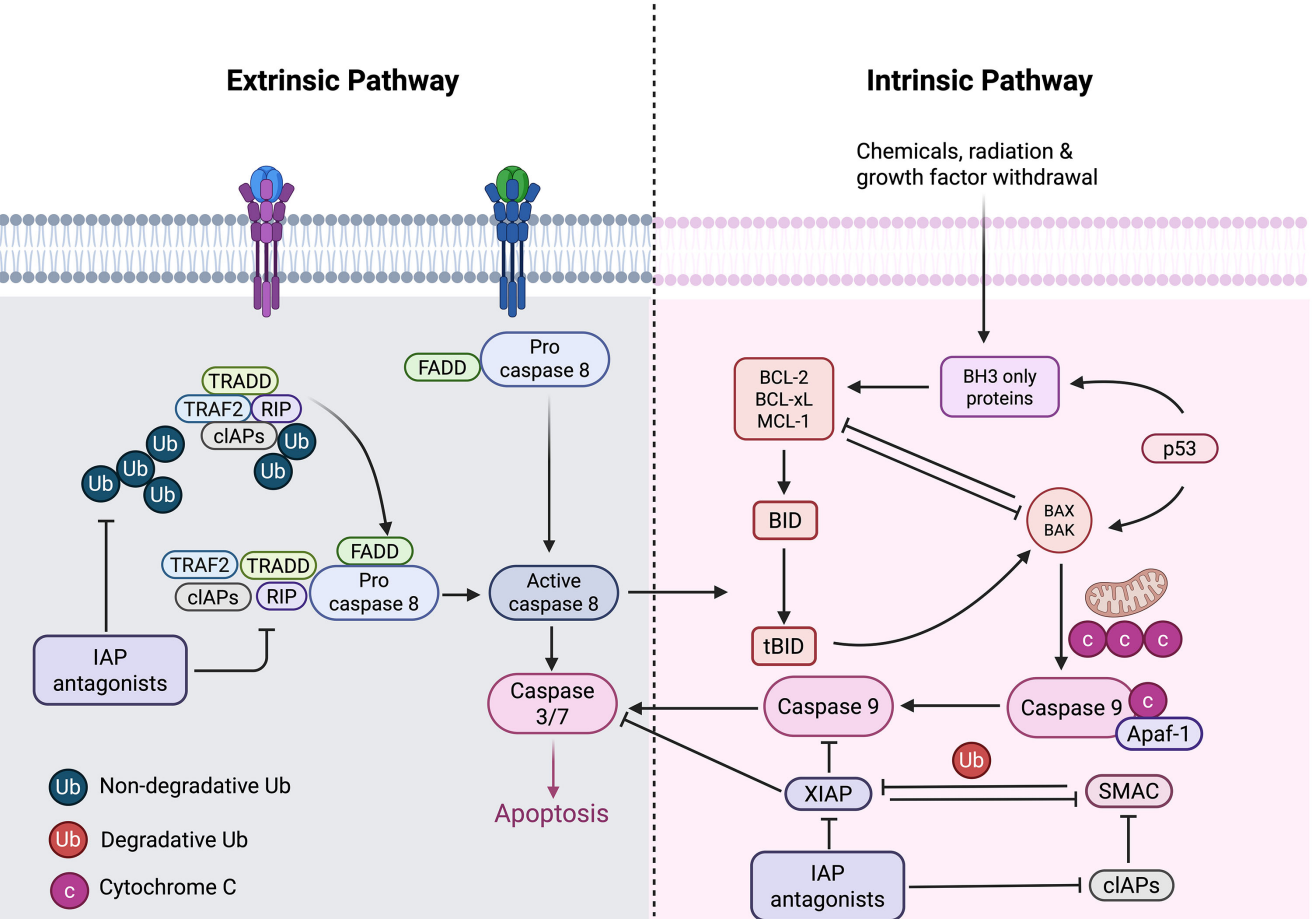
Intrinsic and extrinsic pathways of apoptosis. The intrinsic pathway is initiated by various intracellular stress signals, including chemicals, radiation, and growth factors withdrawal. These signals cause permeation of the outer mitochondrial membrane, resulting in the release of Cyt C from apoptotic precursor proteins into the cell. Subsequently, it forms a complex with Apaf-1 and procaspase-9. Activation of caspase-9, which in turn activates executioner caspases, leading to apoptosis. The intrinsic pathway is regulated by the balance between pro-apoptotic (BAX, BAK) and anti-apoptotic (BCL-2, BCL-xL, MCL-1) Bcl-2 family proteins. In the extrinsic pathway, the ligand binds to the cell surface receptor, initiating the activation of pro-caspase-8, which then cleaves and activates the executioner proteases (protease-3/7), thereby leading to cell apoptosis. Caspase-8 activation can be modulated by the presence of inhibitor of apoptosis proteins (IAPs), which can either be non-degradative (Ub) or degradative (Ub).

Apoptosis is referred to as programmed cell death, a mechanism that does not trigger inflammatory responses and occurs autonomously, thus, eliminating unwanted cells within an organism and maintaining homeostasis. Cell apoptosis mainly has two pathways, (i) endogenous (intrinsic) cell apoptosis, and (ii) exogenous (extrinsic) cell apoptosis (41, 42), as illustrated in the **Figure 2**.

Endogenous apoptosis is activated by various stress conditions, and primarily mediated by the anti-apoptotic protein Bcl-2 and the pro-apoptotic proteins Bax and Bak, while exogenous apoptosis is initiated by the activation of death receptors on the cell membrane, such as the Fas cell surface death receptor (FAS/CD95/APO-1) and TNF receptor superfamily member 1A (TNFRSF1A), which are activated upon binding to their cognate ligands. Both of these pathways mediate the release of cytochrome c (Cyt C) (43), making mitochondrial release of Cyt C an important feature of apoptosis (44, 45). Cyt C is first released from the damaged mitochondria, triggering the activation of cytosolic caspase-3 by forming a complex containing Cyt C, Apaf-1 and caspase-9, thus, leading to cell apoptosis (46). Bcl-2, on the other hand, is an effective inhibitor of apoptosis, which can prevent the destruction of mitochondria and the subsequent release of Cyt C (47–49).

In cardiomyocytes, persistent elevation of intracellular Ca^2+^ can lead to apoptosis, whereby, changes in free Ca^2+^ levels within cardiomyocytes are crucial for the reduction in contractility, thus triggering CaSR activation. Studies have shown that lipopolysaccharide (LPS) can induce increased damage to cardiomyocytes, leading to elevated levels of Cyt C and reduced levels of Bcl-2. Besides mitochondria, Bcl-2 is also localized in the endoplasmic reticulum (ER) membrane, where it confers protection against ER stress (50, 51). Consequently, CaSR activation contributes to myocardial cell injury and apoptosis by upregulating ER stress and autophagy pathways (37).

Studies have shown that mitochondria exhibit obvious Ca^2+^ overload under hypoxic injury conditions, causing mitochondrial damage that eventually leads to apoptosis and necrosis of cardiomyocytes (52–54). The inhibition of Ca^2+^ within mitochondria, thus, effectively reduces cardiomyocyte apoptosis in response to hypoxic injury (55). This suggests that CaSR not only participates in myocardial injury through the ER pathway, but also plays a significant role in cardiomyocytes apoptosis induced by hypertension by regulating mitochondrial dynamics. The research by Lu et al. indicates that after establishing the hypoxia/reoxygenation (H/Re) model, the increase in extracellular Ca^2+^ concentration induces its binding to the CaSR and activates the phospholipase C (PLC) signaling pathway, leading to IP3 accumulation and triggering the release of Ca^2+^ from the sarcoplasmic reticulum (SR) into mitochondria and its concomitant uptake into the mitochondria (16, 56). Activation of CaSR results in mitochondrial dysregulation and aggregation, manifesting as swelling, disarray of cristae, and loss of normal striations. The CaSR is activated by Ca^2+^ and Mg^2+^ (type I activators) (57, 58), which are present in extracellular fluid. In addition to endogenous ions, there are also some exogenous small molecule regulators that can act as regulatory factors for CaSR. The calcimimetic agents such as Calhex231 can inhibit the effect of Ca^2+^ on CaSR (59–62). In the presence of type I CaSR activators, the specific inhibitor of CaSR, Calhex 231, can inhibit CaSR activation induced by Ca^2+^ by binding to the 7TM domain of CaSR that is distant from the Ca^2+^ orthosteric binding site (63). The action modes of different types of ligands on CaSR are illustrated in **Table 1**.

**Table 1:** The typical ligands and action modes of the CaSR.

Type	Representative ligand/compound	Mode of action	Explanation	Reference
Type I agonists	Ca²^+^, Mg²^+^	Orthosteric agonists	Directly linked to the extracellular domain of CaSR, it causes a conformational change in the receptor and activates the signaling pathway.	(57)
Type II positive allosteric modulators, calcimimetics	Cinacalcet, R-467, R-568	Positive allosteric modulator	It does not bind to the native binding site, but binds to the allosteric site to enhance the activation effect of Ca²^+^ on CaSR.	(61)
Type II negative allosteric modulators, calcilytics	Calhex231, NPS-2143	Negative allosteric modulator	Combining with the receptor allosteric site, inhibiting the Ca²^+^-activated effect and reducing the stimulation of CaSR	(62)
Other small molecule regulators	Aminoglycosides, Polyamines (e.g., Spermine)	Positive regulation	The ability to enhance the activation of CaSR suggests that it may affect the activity of CaSR under certain conditions.	(58)

Calhex231 can increase the expression of OPA1 (Optic Atrophy 1) and MFN1 (Mitofusin 1), and reduce the expression of DRP1 (Dynamin-Related Protein 1) (64). Increased cytoplasmic Ca²^+^ stimulates the DRP1-mediated fragmentation of mitochondria, facilitating the translocation of DRP1 to mitochondria (65, 66). However, whether long-term inhibition of fission can exert a protective effect on the heart still requires further validation. Some studies have already attempted to explore this issue by modifying the DRP1 gene in animal models and using DRP1 inhibitors (such as Mdivi-1) (67–69), but the available evidence is still insufficient, suggesting that this is an important direction that requires further in-depth exploration in future research.

Additionally, CaSR activation has been communicated to stimulate calcium-dependent pathways, such as Ca^2+^/calmodulin-dependent protein kinase II (CaMKII) and the calcitonin pathways, thereby, inducing apoptosis in cardiomyocytes (70). CaSR can also promote the proliferation of cardiomyocytes by regulating vascular endothelial growth factor (VEGF), thereby, alleviating cardiomyocyte apoptosis caused by ischemia and hypoxia (71, 72). Of these, VEGF121 and VEGF165 are the main drugs for treating ischemic cardiomyopathy (73). When there is excessive activation or abnormal elevation of calcium load, CaSR can induce cardiomyocyte death through mitochondrial dysfunction and apoptotic signaling pathways (74). However, under conditions of hypoxia without severe reperfusion injury or Ca²^+^ overload, CaSR may promote cell proliferation and survival by upregulating protective factors such as VEGF (74). Thus, it can be seen that the role of CaSR in myocardial injury is highly dependent on specific conditions.

### 3.2 Autophagy

Autophagy is a key degradation process that recycles damaged organelles and long-lived proteins in eukaryotic cells. It helps maintain cellular homeostasis by restoring macromolecules and clearing cytoplasmic debris (14, 37, 75). During autophagy, the substances in the cytoplasm are engulfed by spherical structures characterized by a double-membrane configuration called autophagosomes, and subsequently transported to lysosomes for degradation. Autophagosomes fuse with late endosomes or lysosomes, where their contents are degraded. The resulting products are recycled for macromolecular synthesis and energy metabolism (15, 76).

**Figure 3:**
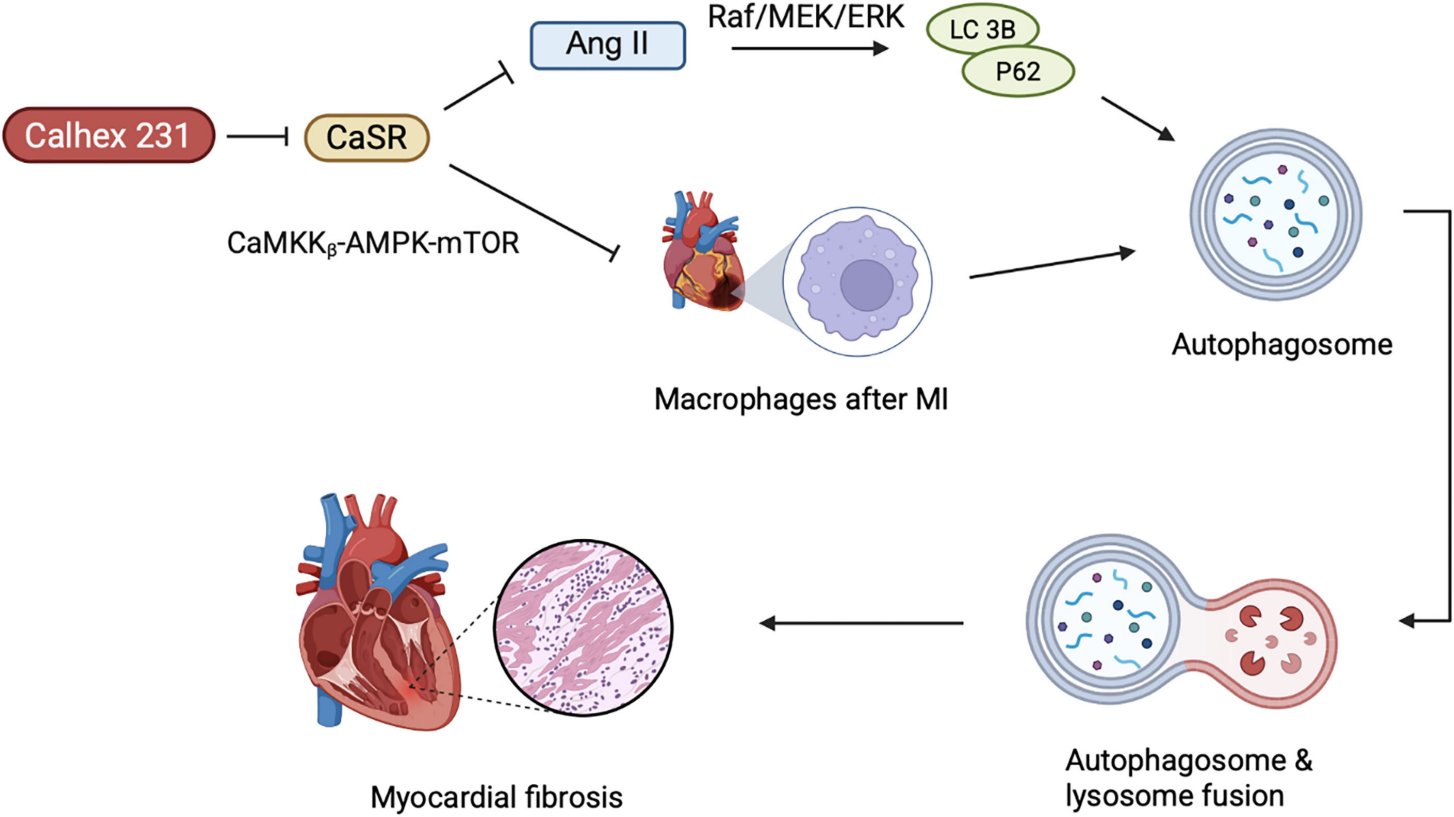
The role of CaSR-mediated autophagy in cardiac remodeling. The activation of CaSR promotes the autophagy process in cardiac fibroblasts, and autophagy further aggravates myocardial fibrosis. AngII can enhance the expression of CaSR and, by activating the Raf/MEK/ERK pathway, upregulates autophagy-related proteins (such as LC3B, P62). After MI, CaSR in macrophages further drives autophagy and activation of the inflammatory body through inhibiting the CaMKK_β_-AMPK-mTOR pathway, thereby exacerbating ventricular remodeling. Calhex 231, as an antagonist of CaSR, can inhibit autophagy and alleviate inflammation, fibrosis, and hypertrophy.

Autophagy is a highly conserved catabolic process that exhibits an intricate association with CVDs, whereby, Ca^2+^ serves as an important messenger molecule for regulating cell death, explicitly autophagic regulation (77), as shown in the **Figure 3**.

Myocardial fibrosis represents one of the pathological hallmarks of diabetic cardiomyopathy. In T1D rats and primary neonatal rat cardiac fibroblasts (CFs), hyperglycemia has been observed to induce myocardial fibrosis (78). Interestingly, the effect of high glucose on CaSR expression varies depending on the cell type. In T1D rats and primary cultured neonatal rat cardiomyocytes, CaSR was noted to be inhibited by high glucose, while in CFs, it leads to excessive proliferation and collagen deposition (79–81). Upon activation in CFs, CaSR leads to Ca^2+^ release followed by activation of autophagy (35), whereby, autophagy further leads to cardiac fibrosis (82–84). Calhex231 can putatively ameliorate cardiac fibrosis by inhibiting autophagy in the cellular system of an organism (82). Furthermore, Angiotensin II (AngII) is implicated in cardiac fibrosis induced by CaSR, leading to cardiac remodeling and upregulation of autophagy in the heart (85), whereby, AngII not only enhances CaSR expression in the myocardial tissues, but also promotes the proliferation and transformation of fibroblasts. Chi et al. reported the onset of AngII mediated cardiac fibrosis, upon CaSR induced autophagy. Meanwhile, the Raf/MEK/ERK signaling pathway may play an important role in AngII-induced autophagy and proliferation of CFs (85). Recent studies have shown that Raf/MEK/ERK signaling pathway regulates autophagy by modulating the expression of key autophagy-related proteins LC3B and P62 (86, 87). Consequently, this leads to proliferation and phenotypic transformation of CFs induced by AngII, which is associated with the upregulation of CaSR-mediated autophagic pathways. However, the specific mechanism still requires further exploration.

Numerous studies have demonstrated increased concentration of Ca^2+^ in the infarcted area of the myocardium after MI, suggesting putative involvement of CaSR in the process of ventricular remodeling after MI. In rat cardiomyocytes, CaSR expression is positively correlated with sensitivity to MI (88). In macrophages, CaSR can increase ventricular remodeling by promoting the activation of NLRP 3 inflammasome (89). Liu et al. revealed Calhex 231 to alleviate myocardial inflammation and fibrosis by inhibiting autophagy of macrophages after MI, inhibiting the activation of NLRP 3 inflammasome and subsequently reducing the release of IL-1β (90). Additionally, the dynamic equilibrium of intracellular Ca^2+^ concentration is an initial factor in myocardial hypertrophy. During myocardial hypertrophy, CaSR mediated autophagy is increased, whereby, elevated Ca^2+^ triggers several Ca^2+^-dependent signaling pathways, culminating into cardiac hypertrophy (91–93). Calhex 231 inhibits autophagy by suppressing CaMKK_β_-AMPK-mTOR pathway, thereby, ameliorating cardiac hypertrophy caused by CaSR activation (82). Simultaneously, activation of CaSR would induce the release of Ca^2+^ from SR of the cardiovascular system (94), and participate in the pathological process of cardiac ischemia/reperfusion injury (95, 96).

### 3.3 Pyroptosis

**Figure 4:**
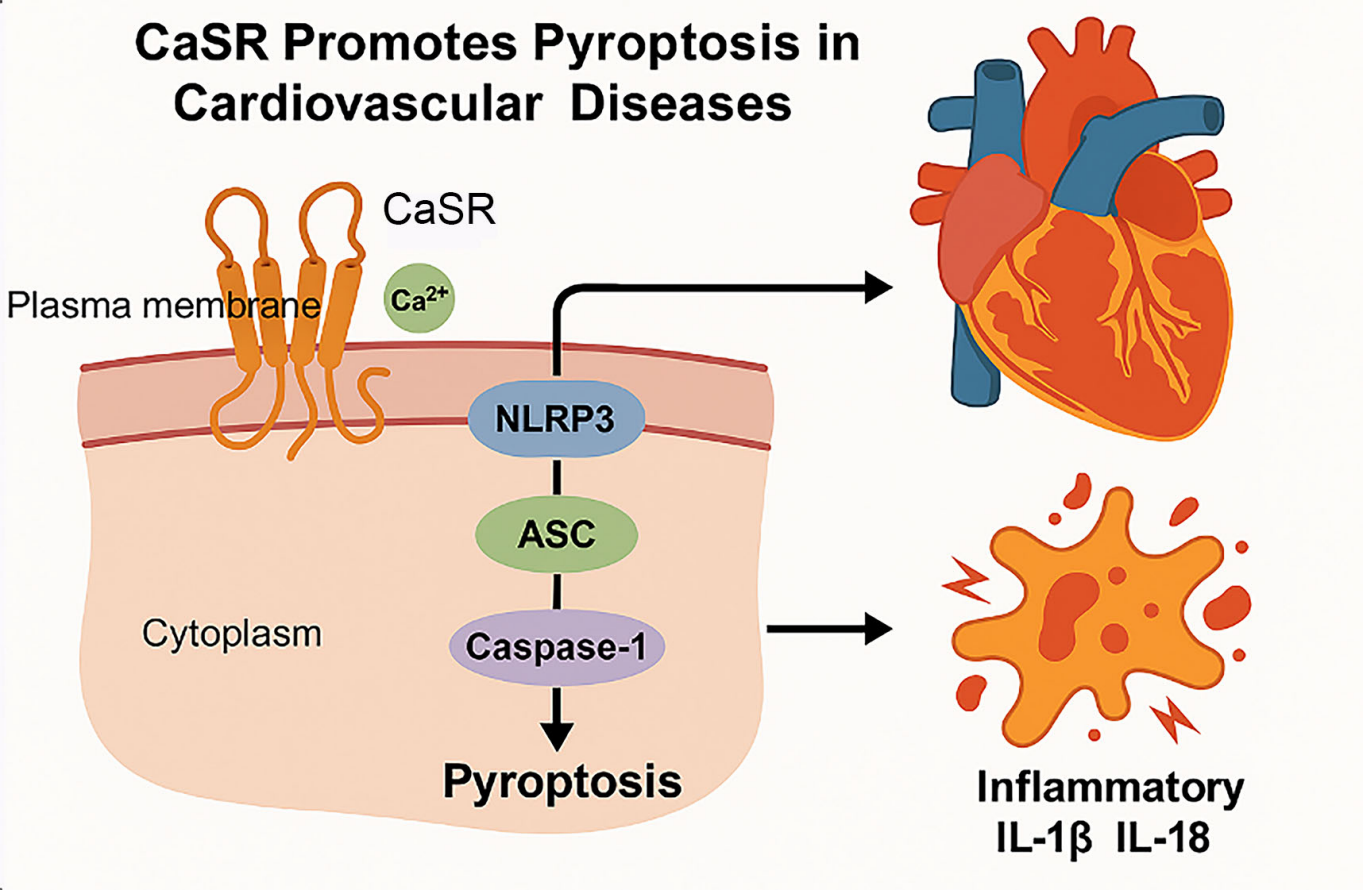
Mechanism of CaSR mediated pyroptosis in CVDs. Cell pyroptosis is a highly inflammatory form of programmed cell death. First, the CaSR located on the plasma membrane is activated by the extracellular Ca^2+^. Upon activation, CaSR triggers the assembly of the NLRP3 inflammasome. And then, the NLRP3 inflammasome recruits ASC (Apoptosis-associated speck-like protein containing a CARD) and pro-caspase-1, leading to the cleavage and activation of caspase-1. Finally, the activation of caspase-1 results in the execution of pyroptosis, characterized by cell lysis and the release of inflammatory cytokines. Pyroptosis leads to the release of inflammatory cytokines such as IL-1β and IL-18, which can exacerbate inflammation and contribute to the pathogenesis of CVDs.

Pyroptosis is a type of programmed cell death mode related to inflammation, which leads to cell death by activating the Caspase-1-mediated signaling pathway (97). Pyroptosis of cells is widely involved in the occurrence and development of various diseases such as infectious diseases (98), metabolic diseases (99), CVDs (100), neurological-related diseases (101), and atherosclerosis (100). Caspase-1, the most crucial member of the Caspase family, assembles the inflammatory sensing factors i.e, NLRP3 inflammasome, AIM2 inflammasome, NLRP1 inflammasome, PYRIN inflammasome, and NLRC4 inflammasome that respond to various stimuli during the process of pyroptosis (102). Studies have shown that caspase-1-dependent pyroptosis induced by NLRP3 inflammasome activation is involved in ischemia/reperfusion (I/R) injury (103). CaSR regulates the generation of NLRP3 inflammasome through Ca^2+^ and cAMP (104), thus, upregulating the expression of ASC, GSDMD, pro-caspase-1, active caspase-1 (P10), IL-1β, and IL-18, thus, exacerbating pyroptosis of cardiomyocytes under hypoxia/reoxygenation (H/R) conditions (105), as illustrated in the **Figure 4** below.

### 3.4 Cell proliferation

In CVDs, the occurrence of various pathological conditions may lead to the loss of control over cell proliferation. Therefore, the research on the regulatory mechanism of cell proliferation is particularly imperative.

In DCM, myocardial fibrosis represents a primary pathological hallmark (106). A study has shown that CaSR is expressed in the myocardial tissues of diabetic rats. After treatment with high-concentration glucose, it can be observed that the number of CFs significantly increases (107). CaSR upregulation in CFs may lead to an increase in intracellular Ca^2+^, and further activate the TGF-β1/SMADs pathway, promoting the proliferation and activation of fibroblasts, ultimately resulting in myocardial fibrosis (108).

Zhong et al. investigated CaSR activation in a diabetic model to increase intracellular Ca^2+^ concentration in VSMCs, enhancing the activity of cystathionine-γ-lyase (CSE) and elevating endogenous H_2_S levels by altering the phosphorylation of CaMK II, which subsequently inhibits VSMCs proliferation (109). This process is associated with the PLC-IP3 receptor, calmodulin (CaM) signaling pathway, and ERK 1/2-dependent signaling pathways (110). In addition, studies have shown that the inhibition of extracellular signal-regulated kinase/mitogen-activated protein kinase (ERK/MAPK) mediated by MFN2 leads to cell cycle arrest (111–113). Zhang et al. suggested that CaSR inhibition might partially attenuate the proliferation of VSMCs by suppressing mitochondrial fission (114).

Therefore, CaSR may regulate VSMCs proliferation through mitochondrial dynamics and ERK signaling pathways, providing a molecular basis for targeted intervention, but the mechanism still needs to be further studied and elucidated. CaSR can be used as a potential therapeutic target for DCM fibrosis and plausibly provide new ideas for the treatment of DCM.

### 3.5 Cell migration

Cell migration is one of the integral cellular process that plays an important role in physiological processes involved in disease pathogenesis such as CVDs. Based on the current landscape, CaSR is implicated in the process of cellular migration, and therefore, the current review also delineates the functional significance of CaSR in cellular migration within the context of CVDs.

I/R injury is a common aspect of CVDs which mediates the expression of high mobility group box-1 protein (HMGB1) and CaSR, thus, affecting cell migration and apoptosis through autophagy, leading to the upregulation of angiogenesis and apoptosis in the late stage (115). The migration of VSMCs also plays a pivotal role in the initiation and progression of intimal thickening associated with atherosclerotic lesions (116). Liu et al. demonstrated that reduced expression of NOTCH3 promotes the migration of VSMCs. Surprisingly, a high expression of CaSR was detected in NOTCH3 siRNA VSMCs, whereby, NOTCH3 knockout leads to a transition of VSMCs from a contractile to a synthetic phenotype, promoting migration. Therefore, CaSR may serve as a potential target for the treatment of atherosclerosis and vein grafting (117). High sugar treatment has also been indicated to enhance the migration and proliferation of CFs; however, this effect is inhibited upon treatment with Calhex 231, a specific inhibitor of CaSR. Calhex 231 alleviates the expression of TGF-β1/Smads pathway by decreasing intracellular Ca^2+^ and inhibiting Itch-ubiquitin proteasome in cardiomyocytes. It inhibits the migration and proliferation of CFs, and also reduces the deposition of collagen, thereby, alleviating glucose-induced myocardial fibrosis (78).

Although the triggers and cell types vary, they all involve changes in intracellular Ca^2+^ signals, which lead to the activation of CaSR. Eventually, they affect key signaling pathways (such as autophagy, TGF-β1/Smads.) to regulate cellular behaviors. Therefore, CaSR plays an important role in cell migration in different CVDs contexts, and is an important target for CVDs treatment.

### 3.6 Cell differentiation

Vascular calcification has been reported to increase the prevalence and mortality rate of CVDs (118). Pertinently, CaSR has also been implicated in vascular repair and maintenance of vascular integrity. Reduced expression of CaSR has been observed in calcified vessels (119). The normal expression of VSMCs receptors is crucial for preventing vascular calcification (120). The hydroxyapatite (HA) identified in these deposits is the same calcium polymorph present in bone, indicating that the calcification process may share mechanisms with bone formation. The transformation of VSMCs into osteoblast-like cells is a crucial mechanism in the progression of vascular calcification (121). Research has found that HA mineral deposits can stimulate the expression of BMP-2 through CaSR, whereby, BMP-2 pathway regulates the differentiation of VSMCs into osteoblast-like cells through SMAD-5 signaling (122).

Embryonic stem cells (ESCs) are multipotent cell lines that can potentially differentiate into distinct cell types (123). ESCs can differentiate into cardiomyocytes and may plausibly be employed in managing heart diseases through cell transplantation. Sun et al. found that CaSR protein exists in mouse embryonic stem cells (mESCs) and mESC-derived cardiomyocytes (mESC-CMs) (124). CaSR activation can increase intracellular Ca^2+^ concentration through the G-PLC-IP3 signal transduction pathway, participating in the differentiation of mESCs into cardiomyocytes through expressional modulation of NKx2.5 and GATA-4 (124). ESC-derived cardiomyocytes have the potential to replenish myocardial loss occurring in MI and other CVDs, thereby, providing new research directions for the treatment of CVDs.

## 4 Role of CaSR in cardiovascular cells

CaSR is expressed in various cardiovascular cell types, where it plays a significant role in modulating key cellular functions that contribute to both physiological regulation and disease progression. Its activity in endothelial cells, VSMCs, cardiomyocytes, and fibroblasts influences processes such as contractility, inflammation, remodeling, and calcification, as detailed below.

### 4.1 Endothelial cells

CaSR regulates vascular tone by influencing nitric oxide (NO) production and endothelial nitric oxide synthase (eNOS) activity in the endothelial cells. Activation of CaSR has been associated with improved endothelial function under certain conditions, while in others, it promotes oxidative stress and inflammation, highlighting its context-dependent function. CaSR also modulates endothelial permeability and angiogenesis, critical for vascular repair and remodeling (125, 126).

### 4.2 Vascular smooth muscle cells

In VSMCs, CaSR contributes to vascular homeostasis by regulating intracellular calcium levels, proliferation, migration, and phenotype switching. In pathological conditions such as hypertension and atherosclerosis, CaSR activation has been linked to increased VSMCs proliferation and calcification, contributing to vascular stiffness and plaque development (127). CaSR can be activated when the extracellular Ca²^+^ concentration is increased, and the PLC is promoted to generate IP3 through G_q/11_ coupling, while the MEK 1/ERK 1,2 pathway is activated. By continuously driving the proliferation of VSMCs, it leads to thickening of the middle layer of the blood vessel wall, narrowing of the lumen and increased stiffness, and exacerbates the progression of hypertension (114, 128).

However, in some models, CaSR appears to exert protective effects by preventing osteogenic differentiation of VSMCs. CaSR is a key binding partner of vasoconstriction-inhibiting factor (VIF) peptide and exerts a protective effect on vascular calcification through its activation (129). VIF inhibited the expression of osteogenic differentiation markers such as BMP2, MSX2, SOX9, and OCN through CaSR. In VSMCs, CaSR activation inhibits calcium influx, ROS production, inflammation, and apoptosis, thereby preventing calcification (129–131). CaSR has dual function and cell state-specific effects, especially in preventing osteogenic differentiation of VSMCs.

The activation of CaSR may result in different and even opposite outputs in the vascular system and the skeletal system, which is an important feature called ‘cell-state specificity’ and ensures the safety of the treatment. Therefore, CaSR therapy is expected to achieve the inhibition of ectopic vascular calcification without affecting physiological bone formation, but the molecular mechanism remains unclear and requires further exploration.

### 4.3 Cardiomyocytes

In cardiomyocytes, CaSR is expressed at both fetal and adult stages, where it modulates intracellular calcium handling, contractile function, and cell survival. CaSR activation has been implicated in myocardial hypertrophy, apoptosis, and I/R injury. The research by Hong et al. indicates that excessive stimulation of calcium-sensitive receptors may exacerbate myocardial cell damage under stress conditions, manifesting as mitochondrial dysfunction and increased cell apoptosis (64). The research conducted by Zhang et al. indicates that moderate activation of CaSR can exert protective effects, including maintaining myocardial contractility and alleviating myocardial fibrosis (132, 133). This indicates that the role of CaSR in the myocardium may be time-dependent and dose-dependent: short-term, moderate activation may maintain cellular function through protective pathways, while long-term or excessive activation may induce pathological signals, leading to cell damage.

### 4.4 Cardiac fibroblasts

CaSR influences ECM remodeling and fibrotic responses in CFs, whereby, it regulates fibroblast activation, proliferation, and collagen synthesis, crucial to the development of cardiac fibrosis following injury. Moreover, dysregulated CaSR signaling in these cells can lead to excessive ECM deposition, contributing to myocardial stiffness and impaired cardiac function (134, 135).

### 4.5 Adipocytes

Within the cardiovascular environment, immune cells have been reported to express CaSR, which may influence local inflammatory responses (136), although this niche still remains underexplored.

This pro-inflammatory effect of CaSR may also affect adipose tissue, which is a key factor in metabolic cardiovascular disease. According to new research, CaSR’s effects are not limited to classical immune cells, but are also found in adipocytes in perivascular adipose tissue (PVAT) (137). CaSR activates and promotes adipocyte predifferentiation, adipogenesis, and adipocyte differentiation (137), while inhibiting lipolysis (138). Studies have shown that activation of CaSR in adipocytes promotes the release of pro-inflammatory adipokines such as leptin and resistin and inhibits adiponectin secretion, leading to metabolic dysfunction in the form of insulin resistance and dyslipidemia (139, 140). There is growing evidence that activation of calcium-sensitive receptors in adipocytes exacerbates inflammation and metabolic dysfunction, leading to atherosclerosis (141, 142). For example, in human and mouse adipocytes, stimulation of calcium-sensitive receptors promote a pro-inflammatory phenotype manifested by increased secretion of cytokines such as IL-6 and MCP-1, while reducing anti-inflammatory adiponectin levels (143). This transition creates a systemic inflammatory environment that accelerates vascular dysfunction. These findings link CaSR in adipocytes to novel mechanisms between metabolic disorders and cardiovascular inflammation, suggesting that modulating CaSR activity in adipose tissue may be a potential strategy to mitigate obesity-related atherosclerosis.

Overall, CaSR exhibits complex and cell-type-specific roles in cardiovascular biology. Its ability to influence diverse signaling pathways and cellular behaviors underscores its importance in cardiovascular health and disease, as indicated in **Table 2**.

**Table 2:** Role of CaSR in different cardiovascular cell type.

Cell type	CaSR expression	Primary functions regulated by CaSR	Relevance in cardiovascular disease
Endothelial Cells	Moderate to High	- Nitric oxide (NO) production- Vascular tone- Angiogenesis- Barrier integrity	- Endothelial dysfunction in hypertension and atherosclerosis- Inflammation and oxidative stress
Vascular Smooth Muscle Cells (VSMCs)	High	- Calcium influx-Proliferation & migration-Phenotype switching-Calcification	- Contributes to vascular remodeling and stiffness- Promotes or inhibits vascular calcification depending on context
Cardiomyocytes	Moderate	- Contractility-Intracellular calcium handling- Hypertrophic signaling- Apoptosis	- Involved in myocardial hypertrophy- Exacerbates ischemia- reperfusion injury when overactivated
Cardiac Fibroblasts	Moderate	- Activation and proliferation- Collagen production- Extracellular matrix (ECM ) remodeling	- Promotes myocardial fibrosis- Contributes to stiffening in heart failure
Macrophages / Immune Cells	Variable (Upregulated in inflammation)	- Cytokine secretion-Chemotaxis- Inflammatory signaling	- Involved in plaque instability in atherosclerosis- Mediates inflammation post- infarction

## 5 Role of CaSR in specific CVDs

CaSR plays diverse cell-specific roles in the pathogenesis of several CVDs. Emerging evidence suggests that altered CaSR expression contributes to the development and progression of cardiovascular conditions such as hypertension, atherosclerosis, cardiac hypertrophy, MI and vascular calcification. However, its function appears to be highly context-dependent, with both protective and deleterious effects reported across various disease models (136).

### 5.1 Hypertension

A growing body of research has proven that RAAS is associated with the occurrence of hypertension caused by diabetes (144). CaSR is not only expressed in the heart, but also in the kidneys (145). Research indicates that reduced CaSR expression contributes to the development of primary hypertension by activating cAMP pathway and the renin-angiotensin system (RAS) (146). cAMP stimulates renin secretion (147), and a significant increase in renin secretion triggers an increase in Ang II and aldosterone, which leads to an increase in blood pressure. CaSR can increase intracellular Ca^2+^ concentration and reduce intracellular cAMP levels, thereby playing an important role in reducing blood pressure (148, 149).

Plasma renin values can directly reflect the degree of renin-mediated vasoconstriction. Studies have shown that CaSR in VSMCs plays a very important role in maintaining blood pressure levels within physiological limits, thus reducing hypertension (150). In VSMCs, CaSR modulates intracellular calcium levels, influencing vasoconstriction and vascular reactivity. Animal studies have shown that CaSR activation can lower blood pressure by promoting vasodilation and natriuresis. Conversely, impaired CaSR signaling may contribute to salt-sensitive hypertension (151). Clinical data on calcimimetics such as cinacalcet suggest potential antihypertensive effects, though their use remains limited in cardiovascular practice.

### 5.2 Atherosclerosis

CaSR is expressed in endothelial cells, macrophages, and VSMCs, which contribute to atherogenesis. In endothelial cells, CaSR may promote nitric oxide (NO) production and preserve barrier integrity under physiological conditions. However, in inflammatory environments, CaSR activation may exacerbate oxidative stress and cytokine release. In VSMCs and macrophages, CaSR appears to influence plaque stability by modulating cell migration, foam cell formation, and calcification. Notably, upregulation of CaSR in atherosclerotic plaques has been reported, suggesting its involvement in disease progression (125, 126, 132).

### 5.3 Cardiac hypertrophy and heart failure

Cardiomyocyte CaSR activity has been linked to pathological hypertrophy and apoptosis. Overactivation of CaSR can lead to increased intracellular Ca^2+^ levels, promoting pro-hypertrophic signaling through calcineurin/NFAT and MAPK pathways. Studies in rodent models indicate that CaSR inhibition may attenuate cardiac remodeling and fibrosis following pressure overload or neurohormonal stimulation. Additionally, CaSR may influence CFs, contributing to ECM deposition and myocardial stiffness (152).

### 5.4 Myocardial ischemia and infarction

During I/R injury, CaSR activation has been associated with calcium overload, mitochondrial dysfunction, and cell death. Inhibition of CaSR in cardiomyocytes during reperfusion has shown protective effects, as it reduced infarct size and improved cardiac function. These findings suggest a detrimental role for CaSR in acute ischemic injury, likely mediated through its influence on calcium signaling and oxidative stress (153).

### 5.5 Vascular calcification

CaSR plays a critical role in regulating mineral metabolism, and its dysregulation is closely linked with vascular calcification, particularly in patients with chronic kidney disease (CKD). In VSMCs, loss of CaSR function promotes osteogenic differentiation, matrix vesicle release, and calcium phosphate deposition. Restoration of CaSR activity via calcimimetics has been shown to inhibit vascular calcification in experimental models and clinical studies, highlighting its potential therapeutic relevance (154).

Comprehensively, CaSR contributes to the pathophysiology of multiple CVDs through diverse, cell-specific mechanisms (36, 64, 70, 150, 153, 155–159), as depicted in **Table 3**. Understanding these disease-specific roles is essential for developing CaSR-targeted therapies tailored to specific cardiovascular problems.

**Table 3:** Summary of studies on CaSR in specific cardiovascular diseases.

Cardiovascular Disease	Study Model/System	Key Findings	Reference
Hypertension	CaSR knockout mice	CaSR deletion leads to increased blood pressure via impaired vasodilation and sodium handing	(150)
	Calcimimetic- treated rats	Cinacalcet reduces blood pressure and improves vascular reactivity	(155)
Atherosclerosis	ApoE-/- mice	CaSR upregulation in plaques correlates with increased inflammation and calcification	(156)
	Human carotid plaques	Elevated CaSR expression in macrophages and smooth muscle cells	(36)
Cardiac Hypertrophy	Ang II- induced hypertrophy in rats	CaSR activation promotes cardiomyocyte hypertrophy via Ca^2+^/NFAT signaling	(157)
	CaSR antagonist treatment	Calciytics reduce myocardial fibrosis and hypertrophy	(70)
Myocardial Infarction	Rat ischemia- reperfusion model	CaSR inhibition decrease infarct size and cardiomyocyte apoptosis	(153)
	H9C2 cardiomyoblasts	CaSR activation increases oxidative stress and mitochondrial dysfunction	(64)
Vascular Calcification	VSMCs in high- phosphate media	CaSR activation inhibis osteogenic transformation of VSMCs	(158)
	CKD patient samples	Reduced CaSR expression associated with enhanced vascular calcification	(159)

## 6 Clinical significance and therapeutic implications

The application of CaSR modulators has shown potential clinical value. CaSR presents a promising yet complex therapeutic target in CVDs. Pharmacological agents that modulate CaSR activity, namely calcimimetics (agonists) and calcilytics (antagonists), have been explored primarily in the context of bone and mineral disorders, such as hyperparathyroidism. However, emerging evidence suggests these agents may also influence cardiovascular outcomes by modulating CaSR activity in endothelial cells, VSMCs, and cardiomyocytes (152).

Cardiovascular complications are also one of the leading causes of death in CKD patients. Secondary hyperparathyroidism (SHPT) is a common complication of CKD and is closely related to cardiovascular pathologies (160, 161). Calcimimetics drugs (such as sinacarcet) have been approved for the treatment of SHPT, and several studies have shown that they may help treat CVDs by reducing parathyroid hormone (PTH) levels, inhibiting vascular calcification, and improving endothelial function (162, 163). In addition, studies have shown that transient hypocalcemia caused by cinalcium may be a risk of cardiovascular death in a time-dependent model (164). However, the current clinical evidence is limited. Most studies on the cardiovascular effects of CaSR modulators have focused on people with CKD, and results are not entirely consistent. Studies have shown that single nucleotide polymorphisms (SNPs) of CASR alter the response to calcified cinnacalcium and help in the treatment of hypocalcemia in CKD hemodialysis patients (165).

Calcimimetics have been shown to reduce vascular calcification and lower blood pressure in some experimental models, while calcilytics may attenuate pathological cardiac remodeling and fibrosis. Despite these promising findings, the systemic activation or inhibition of CaSR can lead to adverse effects due to its widespread tissue distribution and diverse functions. This highlights the need for tissue-selective modulators or targeted drug delivery systems to minimize off-target effects. Currently, clinical data on the cardiovascular effects of CaSR modulators remain limited and inconclusive. While calcimimetics show promise in reducing vascular calcification, the current cardiovascular trial data remain limited, and concerns such as hypocalcemia still persist. Further preclinical and translational studies are essential to validate CaSR as a viable therapeutic target in CVDs and to guide the safe and effective use of its modulators in clinical settings (18, 166).

Additionally, understanding individual genetic variations in CaSR may inform personalized therapeutic strategies. Epidemiological studies have shown that calcium kidney stones are related to polymorphisms in the regulatory region of CaSR genes. rs6776158 located within promoter-1, rs1501899 located in intron 1, and rs7652589 in the 5’-untranslated region were found to reduce the transcriptional activity of promoter-1, and activation of rs1042636 polymorphisms was associated with calcium kidney stones and hypercalciuria (167–170). Gene polymorphisms that reduce CaSR expression may impair the protective effect of CaSR on calcium phosphate and oxalate precipitation, making it more likely to cause kidney stones, while activating the polymorphism rs1042636 may predispose to calcium stones by increasing calcium excretion.

Such variability may be attributed to disease heterogeneity, drug dose differences, and diversity in treatment response due to CaSR gene polymorphisms. Comprehensively, the clinical application of CaSR as a cross-disciplinary therapeutic target still faces challenges. By integrating genetic polymorphism screening with precision drug intervention, the potential of CaSR modulators in the prevention and treatment of CVDs may be maximized.

## 7 Current gaps and contradictory findings

Although numerous studies have highlighted the significance of CaSR in cardiovascular physiology and disease, findings remain inconsistent and sometimes contradictory. In endothelial and VSMCs, CaSR has been shown to both promote and inhibit inflammation and oxidative stress, suggesting a context-dependent role influenced by disease stage or cellular environment. Similarly, its effects on cardiomyocyte hypertrophy and survival vary between *in vitro* and *in vivo* models. A major limitation is the lack of cell-type-specific investigations, leading to conflicting interpretations of systemic versus localized CaSR activation. Moreover, discrepancies in experimental models, CaSR agonists/antagonists used, and dosage regimens contribute to variability in outcomes. The differential impact of CaSR in healthy versus diseased tissues remains poorly understood. These gaps hinder the translation of preclinical data into clinical therapies and underscore the need for more refined models to delineate specific functions of CaSR in cardiovascular pathologies.

## 8 Future directions

Despite significant progress in understanding the role of CaSR in CVDs, several concerns are yet to be addressed. Future research should prioritize the use of cell-type-specific and inducible CaSR knockout models to dissect its precise functions in different cardiovascular tissues. Advanced imaging and biosensing techniques could enable real-time monitoring of CaSR activity and downstream signaling under physiological and pathological conditions. Additionally, investigating the interplay of CaSR cross-talk with other key pathways, such as the renin-angiotensin system and β-adrenergic signaling, may uncover integrative roles in cardiovascular regulation. Likewise, exploring genetic variants of CaSR in patient populations could also provide insight into disease susceptibility and drug responsiveness. Moreover, the development of tissue-targeted CaSR modulators may overcome the limitations of current pharmacological agents and enhance therapeutic precision, at large.

It is worth noting that CaSR also plays a significant role in various physiological and pathological processes outside the cardiovascular system. For instance, adipose tissue not only serves as an energy storage organ, but its secreted adipokines (such as adiponectin and leptin) can affect cardiovascular homeostasis through the endocrine pathway. The high expression of CaSR in adipocytes can regulate adipocyte differentiation and lipid metabolism balance by sensing local calcium signals (such as calcium transient produced by fat breakdown), thereby causing metabolic disorders and further exacerbating atherosclerosis through the “metabolic inflammation” pathway. Additionally, the heart and kidneys form a “heart-kidney axis” through the neuro-humoral network, and their imbalance is the core mechanism of heart failure and the progression of CKD. A significant increase in renin secretion leads to excessive activation of the RAS, and CaSR can inhibit renin release to lower blood pressure and reduce the cardiac load. Therefore, CaSR can serve as a therapeutic target for combined heart-kidney diseases.

Screening patients through CaSR genotyping can achieve accurate selection of therapeutic drugs (such as calcium channel blockers, RAS inhibitors, calcium-sensitive receptor modulators), and predict the risk of adverse reactions (such as hypocalcemia and hyperkalemia), significantly improving the safety and efficacy of the treatment.

Overall, a deeper mechanistic understanding of the dual behavior of CaSR is crucial in order to translate basic findings into effective clinical interventions for CVDs.

## 9 Conclusion

**Figure 5:**
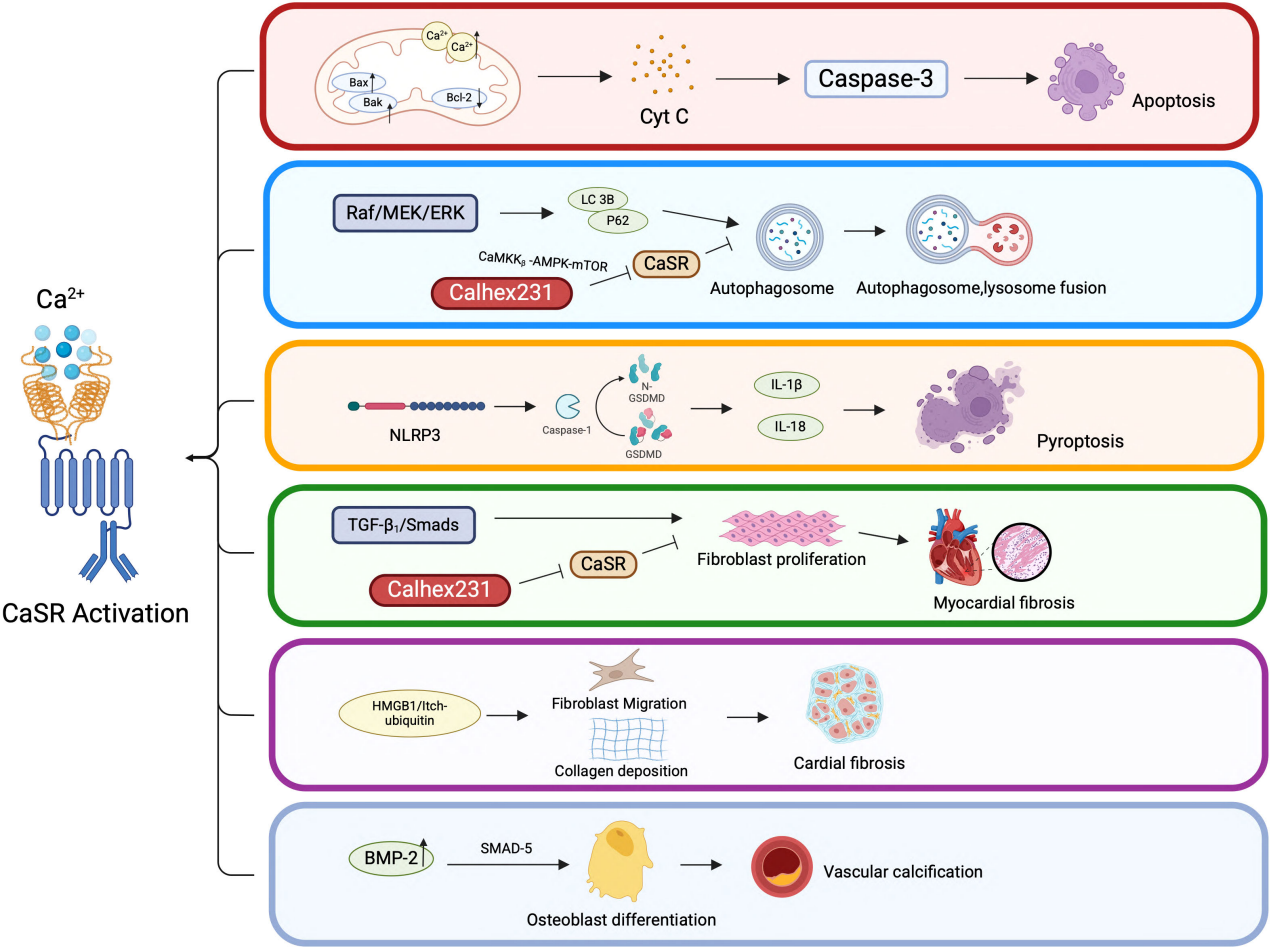
CaSR-mediated cellular behaviors in CVDs. CaSR-mediated cellular behaviors in CVDs. This figure highlights the complex interplay between CaSR activation and various cellular pathways that contribute to CVDs. Understanding these mechanisms is crucial for developing targeted therapies to prevent or treat cardiovascular pathologies associated with CaSR dysregulation.

CaSR is expressed on the surface of various cells/tissues, whereby, it functions by activating distinct signaling pathways by sensing Ca^2+^ concentration in the ECM. The findings of the current review paper revealed significant role of CASR in CVDs, whereby, it modulates various cellular processes such as apoptosis, autophagy, pyroptosis, proliferation, migration and differentiation as depicted in the **Figure 5**. Moreover, intracellular signaling pathways mediated by CaSR can lead to inflammation and fibrosis. Notably, CaSR has demonstrated its clinical relevance as a potential therapeutic target through a range of regulatory functions in CVDs. Therefore, it is imperative to explore specific mechanisms of CaSR function in CVDs and develop novel therapeutic strategies targeting CaSR in future.
